# Limited Trabecular Bone Density Heterogeneity in the Human Skeleton

**DOI:** 10.1155/2016/9295383

**Published:** 2016-04-11

**Authors:** Habiba Chirchir

**Affiliations:** ^1^Department of Biological Sciences, Marshall University, 1 John Marshall Drive, Science Building, Huntington, WV 25755, USA; ^2^Human Origins Program, Department of Anthropology, National Museum of Natural History, Smithsonian Institution, 1000 Constitution Avenue NW, Washington, DC 20560, USA

## Abstract

There is evidence for variation in trabecular bone density and volume within an individual skeleton, albeit in a few anatomical sites, which is partly dependent on mechanical loading. However, little is known regarding the basic variation in trabecular bone density throughout the skeleton in healthy human adults. This is because research on bone density has been confined to a few skeletal elements, which can be readily measured using available imaging technology particularly in clinical settings. This study comprehensively investigates the distribution of trabecular bone density within the human skeleton in nine skeletal sites (femur, proximal and distal tibia, third metatarsal, humerus, ulna, radius, third metacarpal, and axis) in a sample of *N* = 20 individuals (11 males and 9 females). pQCT results showed that the proximal ulna (mean = 231.3 mg/cm^3^) and axis vertebra (mean = 234.3 mg/cm^3^) displayed significantly greater (*p* < 0.01) trabecular bone density than other elements, whereas there was no significant variation among the rest of the elements (*p* > 0.01). The homogeneity of the majority of elements suggests that these sites are potentially responsive to site-specific genetic factors. Secondly, the lack of correlation between elements (*p* > 0.05) suggests that density measurements of one anatomical region are not necessarily accurate measures of other anatomical regions.

## 1. Introduction 

Bone mineral density is generally considered a marker of fracture susceptibility; in other words, low bone mineral density indicates a greater fracture risk (e.g., [1, 2]). There is good correlation between bone mineral density (i.e., amount of bone mineral within a volume), apparent density (i.e., wet weight of bone within a volume), and bone volume fraction (i.e., amount of bone within a volume) with bone mechanical properties (e.g., [3–7]). Mechanical properties of bone show variation that is site-specific thus indicating that bone densities in those particular sites differ depending on the amount of loading (e.g., [8]). Indeed, experimental and observational studies have demonstrated that mechanical loading leads to an increase in trabecular bone density and volume (e.g., [9–15]). The three bone variables referenced above, bone mineral density, apparent density, and bone volume fraction, have been reported extensively in the literature. Although they are technically not the same measures of bone, they quantify aspects of bone that are related, where they estimate either amount of tissue within a region or amount of mineral within a region and thus are indicators of bone strength.

Clinical diagnoses of osteoporosis quantify bone mineral density, often in a few anatomical regions that are safely accessible for sampling. In particular, the femoral neck and lumbar spine are primary diagnostic sites for osteoporosis [16]. Advancements in medical imaging technology have allowed accurate measurement of cortical and trabecular bone density in axial and peripheral sites, such as the distal radius and spine (e.g., [17–19]). With the widespread availability of the quantitative computed tomography and peripheral computed tomography technology, which are capable of separating cortical from trabecular bone within a site, it has been shown that trabecular bone density varies within the human body; however, these reports have mostly concentrated on lumbar vertebrae and the distal radius (e.g., [19, 20]). For instance, Fujii et al., 1996 [20], showed that the lumbar vertebral sites (L2, L3, and L4) have about 75% more density than the distal radius. Other clinical studies that have attempted to characterize trabecular bone microstructure in multiple anatomical sites demonstrated that there are trabecular bone architectural and volumetric differences in different skeletal sites in humans, which may be dependent on loading on a skeletal site (e.g., [21–24]). Specifically, Groll et al., 1999 [21], found that lower limb elements (femur and tibia) were relatively similar compared to the upper limb (radius) in bone mineral density. Amling et al., 1996 [22], showed differences between lumbar vertebrae and the femoral neck, specifically in bone volume fraction with a 7% difference. Hildebrand et al., 1999 [23], found that femoral heads display high bone volume fraction and thick trabeculae compared to the lumbar vertebrae. Ulrich et al. [24] also reported higher bone volume fraction in the femoral head than in the lumbar spine and iliac crest.

Apart from mechanical loading, genetic factors have been implicated in the control of site-specific bone mineral density (e.g., [25, 26]). In fact, recently, genome-wide association studies assessing bone mineral density in the femoral neck and lumbar vertebrae identified 56 loci that explain the phenotypic variance in adult bone mineral density [27, 28]. In summary, these reports show that trabecular bone microstructure, and trabecular bone density, may reflect differences in mechanical loading as well as differences that are systemic. Thus, taken together, these findings raise questions regarding the distribution of trabecular bone density and bone volume fraction throughout the human skeleton in limb elements loaded extensively in locomotion and in elements minimally used in locomotion. They also raise questions on whether one site can accurately be used to predict density in another. This study aims to assess the trabecular bone mineral density (referred to throughout the paper as trabecular density) distribution and site correlation in multiple anatomical sites of the human skeleton.

## 2. Materials and Methods

The skeletal samples studied were derived from the Terry collection. This is an anatomical collection of late 19th and early 20th century Americans of known age curated from the St. Louis hospital and is currently housed at the National Museum of Natural History, the Smithsonian Institution [29]. Adult and nongeriatric individuals were chosen based on age; individuals ranged from ages 25 to 45. A sample of 20 individuals consisting of 11 males and 9 females was selected and trabecular density in the following epiphyses per individual was measured: femoral head, distal tibia, proximal tibia, 3rd metatarsal head (MT3), humeral head, proximal ulna, distal radius, 3rd metacarpal head (MC3), and axis.

**Figure 1:**
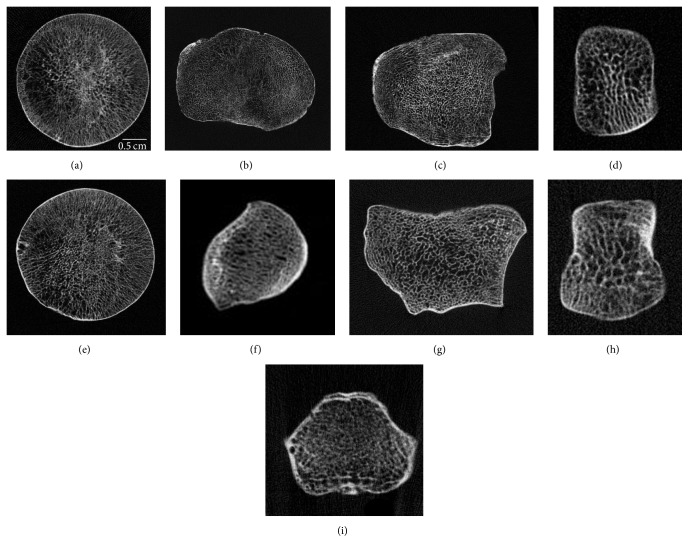
Cross-sectional slices scanned at 100 *μ*m through the (a) femoral head, (b) proximal tibia, (c) distal tibia, (d) MT3 head, (e) humeral head, (f) proximal ulna, (g) distal radius, (h) MC3 head, and (i) axis.

Epiphyseal elements were scanned using a Norland Stratec Research SA-peripheral Quantitative Computed Tomography (pQCT) with inbuilt software version 5.40 (Figures 1(a)–1(i)). Images were scanned at a slice thickness of 100 *μ*m and voxel size of 100 *μ*m, using 50 kV. CALCBD, an inbuilt function used to derive density results within an ROI, was used. Peel Mode 1 with a set threshold of 500 mg/cm^3^ was used to separate trabecular bone from cortical bone. The peel percentage was set between 65% and 75%, depending on the amount of visible cortical bone; that is, the internal 65–75% of the joint was demarcated as trabecular bone, and the remaining part of the joint (outer shell) was treated as cortical bone. Although pQCT was originally designed to measure living bone, the overall bone mineral content is stable and accounts for 62–65% of the dry bone weight [30, 31]. Thus, using pQCT to measure dry bone is valid because the density of the mineral will still be high.

**Figure 2:**
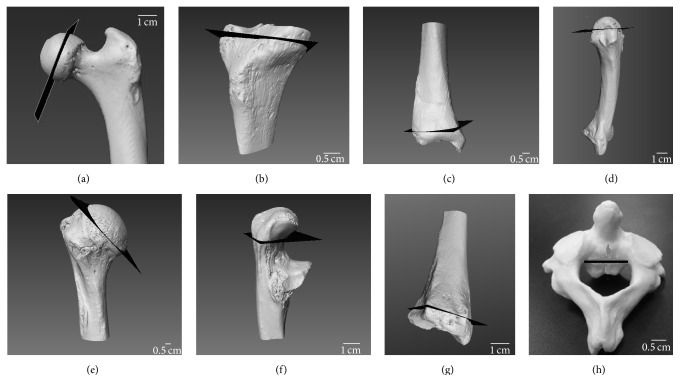
Images showing the location of scanning on the skeletal elements indicated by black slices in pQCT: (a) femoral head, (b) proximal tibia, (c) distal tibia, (d) MT3 and MC3, (e) humerus, (f) proximal ulna, (g) distal radius, and (h) axis.

Elements of interest were placed in the pQCT scanner as follows: the mediolateral breadth of the femoral and humeral heads was estimated as the distance from the lateral and central most point of the head to the medial and central most point. Half of that breadth was identified as the scanning location (Figures 2(a) and 2(e)). On the proximal tibia, the scan was obtained at half of the superior-inferior breadth (Figure 2(b)), that is, the height on the lateral side from the fibular articulation to the edge of tibial plateau The superior-inferior breadth of the epiphysis was measured on the distal tibia, and half of that breadth was identified as the scanning site (Figure 2(c)). On the metacarpal head and metatarsal head, half of the superior-inferior breadth was identified as the location of scanning from the central and superior most point of the head to the epiphyseal line (Figure 2(d)). On the proximal ulna, the superior-inferior breadth was measured as the breadth of olecranon process from the most superior point to the center of the trochlear notch (Figure 2(f)). On the distal radius, half of the depth of the articulation surface with the ulna was identified as the scanning location (Figure 2(g)). Lastly, on the axis, the superior-inferior breadth of the axis body excluding the dens was measured, and half of that height was identified as the location, in which the scan would be obtained (Figure 2(h)).

One-way ANOVA with repeated measures were performed in R version 3.2.2 to identify whether there were statistically significant differences in the trabecular density of the various skeletal elements. Secondly, Pearson's correlation tests were computed between anatomical sites to identify if trabecular density in any of the sites was correlated. Additionally, Student* t*-tests between males and females in the sample did not yield a significant difference and consequently the results were pooled.

## 3. Results

**Figure 3:**
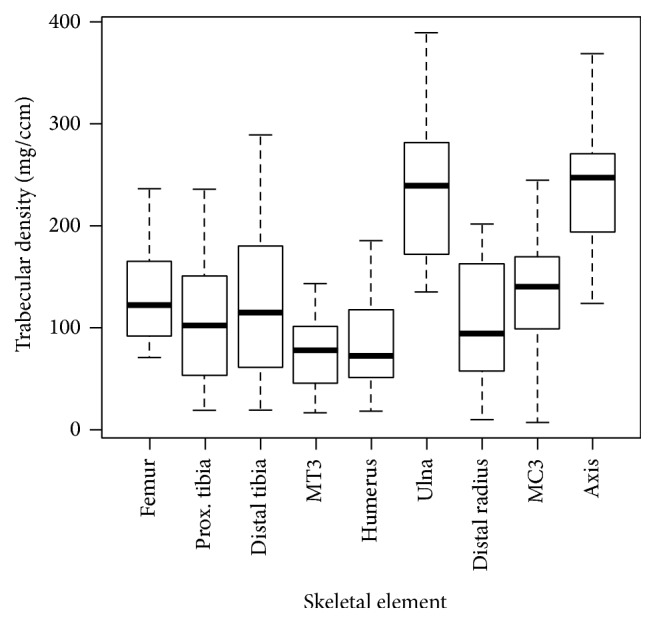
Trabecular bone density in nine skeletal sites showing that the proximal ulna and axis have significantly greater density than all other elements (*p* < 0.01); “prox.” refers to proximal.

Mean trabecular density values with standard deviations and standard errors are presented in Table 1. The proximal ulna and the axis displayed the highest trabecular density values with means of 231.3 mg/cm^3^ and 234.3 mg/cm^3^, respectively. These two elements were significantly greater (*p* < 0.01) than all other elements (Table 2, Figure 3). Furthermore, the axis and proximal ulna were not significantly different from one another (*p* > 0.05). One-way ANOVA of trabecular densities among the rest of the elements, that is, femoral head, proximal and distal tibia, third metacarpal head, humeral head, proximal ulna, distal radius, third metacarpal head, and axis, did not show any significant differences (*p* > 0.05). Lastly, Pearson's correlations did not reveal any statistically significant correlations in any of the elements (*p* > 0.01). Correlations and *p* values are reported in Table 3.

## 4. Discussion

The goal of this study was to assess trabecular density distribution and anatomical site correlation in multiple sites in the skeleton. The study found that two elements, the proximal ulna and axis, had significantly greater trabecular density than all the other elements: femoral head, proximal tibia, distal tibia, MT3 head, humeral head, distal radius, and MC3 head. These elements were fairly consistent in density and did not have significant differences among them. These findings suggest that there is homogeneity of trabecular density in the majority of the elements. Although limited in number of skeletal elements studied and contrary to this study's finds, a number of studies have found that overall trabecular microstructure and bone mineral density [21–23, 32, 33] have considerable heterogeneity in the different sites.

For instance, Hildebrand et al. [23] and Ulrich et al. [24] found that across elements (the 2nd lumbar vertebrae, femoral head, calcaneus, and the iliac crest), there were differences in bone volume fraction, with the femoral head exhibiting the greatest amount of volume and the lumbar the least, despite differences in loading these regions. Groll et al., 1999 [21], found significant differences in upper versus lower limb elements; however, they also reported homogeneity within the lower limb. Specifically, they found significantly different bone volume fraction between the radius and femur, while the tibia displayed similar bone volume fraction to the femur. They concluded that physical activity was responsible for these differences based on the loading difference between upper and lower limbs. Thus, bone measurements of lower limb are better predictors of fracture risk in the lower limb rather than measurements of the upper limb. Based on this study's finds that there is significant homogeneity of trabecular density, using measures of a region such as the lower limb elements to predict other lower limb element fracture risk appears legitimate. However, despite this homogeneity in the majority of elements, this study did not find any statistically significant correlation between any of the elements (Table 3), contrary to Groll et al. [21]. The lack of correlation therefore undermines the use of density measurements from one site as representative measurements of other anatomical regions.

Bone plays a significant biomechanical role during locomotion and is critical for energy absorption in the joints [34, 35]. Trabecular bone increases its stiffness by increasing the amount of bone or by altering the orientation, thickness, number, and spacing of individual trabeculae (e.g., [36]). Because the energy absorbed is proportional to density, denser bone absorbs more energy per unit volume [37, 38]. Thus, it is logical to predict that an individual's physical activity would have effects on trabecular density in the various sites. However, my results show that elements of the lower limb that are under substantive loading during locomotion do not display the greatest trabecular density. Just as articular surfaces are functionally constrained to maintain joint congruity (e.g., [39, 40]), it is possible that there is constrain in growing more trabeculae. Instead of growing more bone, which would be metabolically expensive not only to grow but also to maintain, trabecular bone responds to loading stimuli via alterations in architecture (e.g., degree of anisotropy).

Generally, diaphyses which are primarily composed of cortical bone resist compression, bending, and torsion, whereas joints are primarily resisting compressive loads [41, 42]. Therefore, it is plausible that my results of trabecular density homogeneity in the majority of anatomical sites may not be representative of mechanical loading due to the differences in loads engendered at the joints, but rather, density in cortical bone of the diaphyses would better represent loading differences.

This study did not find any significant differences between males and females, which is consistent with several studies showing no differences in density in the spine when micro-CT were used [33, 43, 44]. However, others have found site-specific differences between males and females (e.g., [21, 45, 46]). The sample studied consisted of healthy individuals under the age of 45 [29] whose bone loss was potentially not significant enough to be observed if any. Although nutrition and exercise are an important part assessing bone density, given the historical nature of the samples, it is beyond the scope of this study to investigate those variables.

Enlarged joint surfaces help distribute forces over a larger surface area (e.g., [47]) as observed in humans. Therefore, one might expect that these joints would exhibit greater trabecular density to support increased loads. However, the results presented here also indicate that even relatively large joints do not exhibit high trabecular density, such as the femoral and humeral heads compared to the proximal ulna and axis. These elements do no display high densities due to the manner in which the large joint surfaces influence bone growth. The large joint surfaces enable the distribution of joint reaction forces over a large area and lead to a decrease in tissue stress and consequently a decrease in bone growth [48]. Small joints such as the axis on the other hand experience tissue stress within a relatively small area and thus bone growth occurs in order to absorb tissue stress. Despite this explanation, other small elements such as the third metatarsal and metacarpal did not display high density, as the above explanation would predict thus suggesting another factor apart from mechanical loading is influencing trabecular density distribution. Additionally, strength in the vertebrae is maintained by trabecular bone and not cortical bone; therefore, to maintain that strength, trabecular bone growth is enhanced.

While the proximal ulna is not loaded during locomotion, this does not discount the fact that it is under loading in flexion and extension when performing manual tasks such as lifting and carrying. Moreover, the elbow joint is a hinge joint and its range of movements is limited, and it only experiences axial loading. In addition, on visual examination, the proximal ulna exhibits dense cortical bone, which along with the high trabecular density buttresses the forces engendered at this joint. Together the complex suite of demands and constraints at this joint presents a challenge in interpreting the results purely from a biomechanical standpoint.

Apart from biomechanical functions, trabecular bone plays other significant roles. It provides nearly 90% of the total bone surface, which represents ~80% of the calcium exchange surface [49]. It is a calcium reserve for up to 70% of total calcium turnover per day [50]. Furthermore, genome-wide association studies assessing bone density in the femur and lumbar vertebrae have shown specific loci that account for ~4-5% of the variation in bone mineral density [28]. Although a small percentage, these loci contribute to variation in mineral density that is site-specific. Lastly, Varanasi et al. [49] also showed that osteocyte, osteoblast, and osteoclast related genes are upregulated in some sites such as the iliac crest, compared to its expression in the spine.

Taken together, the previous reports and the findings from this study indicate that bone composition is homogeneous in some sites and heterogeneous in others and may reflect differences in complex physiological, developmental, and genetic factors that influence bone responses to stimuli differently in the various sites. Given the mechanical and physiological complexity of trabecular bone function, these results are not easily interpreted.

This study presents an assessment of the distribution of trabecular density in the human skeleton; but it has some limitations that cannot be ignored. Those limitations are twofold: (i) the method used is not directly comparable to what some have obtained directly using micro-CT, DEXA, and QCT imaging, some of which account for bone volume fraction and others for bone mineral density; however, the bone variables measured in this study and other studies are related; (ii) it assessed trabecular density in one population (*n* = 20), an industrial one; the observed density patterns may vary according to broad activity patterns.

In conclusion, this study examined the distribution of trabecular density in the human skeleton and the legitimacy of utilizing density values of one site as a representative of another. The results reveal a complex pattern that is both consistent and inconsistent with previous work. They show a pattern among healthy individuals in which few sites have high trabecular density while the majority of sites sampled are somewhat similar, albeit with no obvious correlations. This suggests that these sites are not necessarily accurate indicators of loading and fracture susceptibility in other skeletal sites.

## Figures and Tables

**Table 1:** Mean trabecular density (mg/cm^3^) with standard deviation in the first row in parentheses and standard errors in the second row in parentheses. All samples were *n* = 20.

Femur	Distal tibia	Proximal tibia	MT3	Humerus	Ulna	Distal radius	MC3	Axis
127.15	105.94	118.64	77.55	83.73	231.31	106.03	133.25	234.31
(46.83)	(64.08)	(72.09)	(37.09)	(48.22)	(67.02)	(59.04)	(59.13)	(62.15)
(10.47)	(16.12)	(14.33)	(8.29)	(11.36)	(14.98)	(13.2)	(13.22)	(13.89)

**Table 2:** Only significantly different values are presented. Axial and proximal ulna trabecular density is significantly greater (*p* < 0.01) than other elements.

Femur-axis	Distal tibia-axis	Proximal tibia-axis	MT3 head-axis	Ulna-femur	Ulna-distal tibia	Ulna-proximal tibia	Ulna-MT3 head
<0.001	<0.001	<0.001	<0.0001	<0.001	<0.0015	<0.001	<0.001

Humerus-axis	Distal radius-axis	MC3 head-axis	Ulna-humerus	Ulna-distal radius	Ulna-MC3 head		

<0.001	<0.001	<0.001	<0.001	<0.001	<0.001		

**Table 3:** Pearson's correlation shown as “*r*” and associated *p* values, “prox.” refers to proximal and “dist.” refers to distal.

Femur-prox. tibia	Femur-dist. tibia	Femur-MT3	Femur-humerus	Femur-ulna	Femur-dist. radius	Femur-MC3
*r* = −0.313 *p* = 0.274	*r* = −0.077 *p* = 0.744	*r* = −0.148 *p* = 0.544	*r* = −0.036 *p* = 0.880	*r* = 0.212 *p* = 0.369	*r* = −0.188 *p* = 0.426	*r* = 0.066 *p* = 0.787

Prox. tibia-dist. tibia	Prox. tibia-MT3	Prox. tibia-humerus	Prox. tibia-ulna	Prox. tibia-dist. radius	Prox. tibia-MC3	

*r* = 0.346 *p* = 0.225	*r* = −0.1363 *p* = 0.657	*r* = 0.186 *p* = 0.443	*r* = 0.255 *p* = 0.377	*r* = 0.487 *p* = 0.076	*r* = −0.052 *p* = 0.865	

Dist. tibia-MT3	Dist. tibia-humerus	Dist. tibia-ulna	Dist. tibia-dist. radius	Dist. tibia-MC3		

*r* = 0.344 *p* = 0.148	*p* = 0.572 *r* = 0.133	*p* = 0.802 *r* = 0.059	*p* = 0.651 *r* = 0.107	*p* = 0.593 *r* = 0.130		

MT3-humerus	MT3-ulna	MT3-dist. radius	MT3-MC3			

*r* = 0.133 *p* = 0.572	*r* = 0.240 *p* = 0.321	*r* = −0.006 *p* = 0.980	*p* = 0.299 *r* = −0.258			

Humerus-ulna	Humerus-dist. radius	Humerus-MC3				

*r* = 0.652 *p* = 0.013	*r* = −0.153 *p* = 0.616	*r* = 0.287 *p* = 0.231				

Ulna-dist. radius	Ulna-MC3					

*r* = 0.376 *p* = 0.101	*r* = −0.053 *p* = 0.829					

Dist. radius-MC3						

*r* = −0.0807 *p* = 0.742						

## References

[1] Genant H. K., Engelke K., Fuerst T. (1996). Noninvasive assessment of bone mineral and structure: state of the art. *Journal of Bone and Mineral Research*.

[2] Cummings S. R., Karpf D. B., Harris F. (2002). Improvement in spine bone density and reduction in risk of vertebral fractures during treatment with antiresorptive drugs. *The American Journal of Medicine*.

[3] Currey J. D. (1969). The mechanical consequences of variation in the mineral content of bone. *Journal of Biomechanics*.

[4] Schaffler M. B., Burr D. B. (1988). Stiffness of compact bone: effects of porosity and density. *Journal of Biomechanics*.

[5] Keaveny T. M., Hayes W. C., Hall B. K. (1993). Mechanical properties of cortical and trabecular bone. *Bone Growth*.

[6] Zioupos P., Currey J. D., Casinos A., De Buffrénil V. (1997). Mechanical properties of the rostrum of the whale *Mesoplodon Densirostris*, a remarkably dense bony tissue. *Journal of Zoology*.

[7] Zioupos P., Currey J. D., Casinos A. (2000). Exploring the effects of hypermineralisation in bone tissue by using an extreme biological example. *Connective Tissue Research*.

[8] Lanyon L. E., Rubin C. T. (1984). Static versus dynamic loads an influence on bone remodeling. *Journal of Biomechanics*.

[9] Lanyon L. E. (1974). Experimental support for the trajectorial theory of bone structure. *The Journal of Bone & Joint Surgery—British Volume*.

[10] Biewener A. A., Fazzalari N. L., Konieczynski D. D., Baudinette R. V. (1996). Adaptive changes in trabecular architecture in relation to functional strain patterns and disuse. *Bone*.

[11] Mori T., Okimoto N., Sakai A. (2003). Climbing exercise increases bone mass and trabecular bone turnover through transient regulation of marrow osteogenic and osteoclastogenic potentials in mice. *Journal of Bone and Mineral Research*.

[12] Wallace J. M., Rajachar R. M., Allen M. R. (2007). Exercise-induced changes in the cortical bone of growing mice are bone- and gender-specific. *Bone*.

[13] Barak M. M., Lieberman D. E., Hublin J.-J. (2011). A Wolff in sheep's clothing: trabecular bone adaptation in response to changes in joint loading orientation. *Bone*.

[14] Harrison L. C. V., Nikander R., Sikiö M. (2011). MRI texture analysis of femoral neck: detection of exercise load-associated differences in trabecular bone. *Journal of Magnetic Resonance Imaging*.

[15] Warden S. J., Roosa S. M. M., Kersh M. E. (2014). Physical activity when young provides lifelong benefits to cortical bone size and strength in men. *Proceedings of the National Academy of Sciences of the United States of America*.

[16] Cummings S. R., Black D. M., Nevitt M. C. (1993). Bone density at various sites for prediction of hip fractures. The study of osteoporotic fractures research. *The Lancet*.

[17] Genant H. K., Ettinger B., Harris S. T., Block J. E., Steiger P., Riggs B. L., Melton L. J. (1988). Quantitative computed tomography in assessment of osteoporosis. *Osteoporosis: Etiology, Diagnosis, and Management*.

[18] Butz S., Wüster C., Scheidt-Nave C., Götz M., Ziegler R. (1994). Forearm BMD as measured by peripheral quantitative computed tomography (pQCT) in a German reference population. *Osteoporosis International*.

[19] Grampp S., Lang P., Jergas M. (1995). Assessment of total, trabecular, and cortical bone at radius and spine. *Journal of Bone and Mineral Research*.

[20] Fujii Y., Chikawa T., Nakamura T., Goto B., Fujita T. (1996). Comparison of trabecular bone density at vertebral and radial sites using quantitative computed tomography. *Osteoporosis International*.

[21] Groll O., Lochmüller E.-M., Bachmeier M., Willnecker J., Eckstein F. (1999). Precision and intersite correlation of bone densitometry at the radius, tibia and femur with peripheral quantitative CT. *Skeletal Radiology*.

[22] Amling M., Herden S., Pösl M., Hahn M., Ritzel H., Delling G. (1996). Heterogeneity of the skeleton: comparison of the trabecular microarchitecture of the spine, the iliac crest, the femur, and the calcaneus. *Journal of Bone and Mineral Research*.

[23] Hildebrand T., Laib A., Müller R., Dequeker J., Rüegsegger P. (1999). Direct three-dimensional morphometric analysis of human cancellous bone: microstructural data from spine, femur, iliac crest, and calcaneus. *Journal of Bone and Mineral Research*.

[24] Ulrich D., van Rietbergen B., Laib A., Rüegsegger P. (1999). The ability of three-dimensional structural indices to reflect mechanical aspects of trabecular bone. *Bone*.

[25] Peacock M., Turner C. H., Econs M. J., Foroud T. (2002). Genetics of osteoporosis. *Endocrine Reviews*.

[26] Turner C. H. (2002). Biomechanics of bone: determinants of skeletal fragility and bone quality. *Osteoporosis International*.

[27] Styrkarsdottir U., Halldorsson B. V., Gretarsdottir S. (2008). Multiple genetic loci for bone mineral density and fractures. *The New England Journal of Medicine*.

[28] Estrada K., Styrkarsdottir U., Evangelou E. (2012). Genome-wide meta-analysis identifies 56 bone mineral density loci and reveals 14 loci associated with risk of fracture. *Nature Genetics*.

[29] Hunt D. R., Albanese J. (2005). History and demographic composition of the Robert J. Terry anatomical collection. *American Journal of Physical Anthropology*.

[30] Galante J., Rostoker W., Ray R. D. (1970). Physical properties of trabecular bone. *Calcified Tissue Research*.

[31] Hansson T., Keller T., Spengler D. (1987). Mechanical behavior of the human lumbar spine: II. Fatigue tailure during dynamic compressive loading. *Journal of Orthopaedic Research*.

[32] Thomsen J. S., Ebbesen E. N., Mosekilde L. (2002). Static histomorphometry of human iliac crest and vertebral trabecular bone: a comparative study. *Bone*.

[33] Eckstein F., Lochmüller E.-M., Lill C. A. (2002). Bone strength at clinically relevant sites displays substantial heterogeneity and is best predicted from site-specific bone densitometry. *Journal of Bone and Mineral Research*.

[34] Radin E. L., Orr R. B., Kelman J. L., Paul I. L., Rose R. M. (1982). Effect of prolonged walking on concrete on the knees of sheep. *Journal of Biomechanics*.

[35] Swartz S. M. (1989). The functional morphology of weight bearing: limb joint surface area allometry in anthropoid primates. *Journal of Zoology*.

[36] Rubin C., Turner A. S., Bain S., Mallinckrodt C., McLeod K. (2001). Low mechanical signals strengthen long bones. *Nature*.

[37] Currey J. D. (2002). *Bone: Structure and Mechanics*.

[38] Swartz S. M., Parker A., Huo C. (1998). Theoretical and empirical scaling patterns and topological homology in bone trabeculae. *Journal of Experimental Biology*.

[39] Pauwels F. (1976). *Biomechanics of the Normal and Diseased Hip*.

[40] Lieberman D. E., Devlin M. J., Pearson O. M. (2001). Articular area responses to mechanical loading: effects of exercise, age, and skeletal location. *American Journal of Physical Anthropology*.

[41] Carter D. R., Spengler D. M. (1978). Mechanical properties and composition of cortical bone. *Clinical Orthopaedics and Related Research*.

[42] Rubin C. T., Lanyon L. E. (1984). Regulation of bone formation by applied dynamic loads. *The Journal of Bone & Joint Surgery—American Volume*.

[43] Lochmüller E.-M., Eckstein F., Kaiser D. (1998). Prediction of vertebral failure loads from spinal and femoral dual-energy X-ray absorptiometry, and calcaneal ultrasound: an in situ analysis with intact soft tissues. *Bone*.

[44] Cheng X. G., Nicholson P. H. F., Boonen S. (1997). Prediction of vertebral strength in vitro by spinal bone densitometry and calcaneal ultrasound. *Journal of Bone and Mineral Research*.

[45] Bouxsein M. L., Melton L. J., Riggs B. L. (2006). Age- and sex-specific differences in the factor of risk for vertebral fracture: a population-based study using QCT. *Journal of Bone and Mineral Research*.

[46] Eckstein F., Matsuura M., Kuhn V. (2007). Sex differences of human trabecular bone microstructure in aging are site-dependent. *Journal of Bone and Mineral Research*.

[47] Ruff C. B. (2002). Long bone articular and diaphyseal structure in old world monkeys and apes. I. Locomotor effects. *American Journal of Physical Anthropology*.

[48] Cotter M. M., Loomis D. A., Simpson S. W., Latimer B., Hernandez C. J. (2011). Human evolution and osteoporosis-related spinal fractures. *PLoS ONE*.

[49] Varanasi S. S., Olstad O. K., Swan D. C. (2010). Skeletal site-related variation in human trabecular bone transcriptome and signaling. *PLoS ONE*.

[50] Parfitt A. M., Recker R. (1983). The physiological and clinical significance of bone histomorphometric data. *Bone Histomorphometry: Techniques and Interpretations*.

